# An Innovative University and Non-Governmental Organization Partnership in Global Health: Application of the Brocher Declaration to 20 Years of Collaboration in Kenya

**DOI:** 10.5334/aogh.5383

**Published:** 2026-09-16

**Authors:** Marie H. Martin, Ash Rogers, Joseph Starnes, Elizabeth S. Rose, Harriett Myers, Julius Mbeya, Milton Ochieng’, James Nardella, Katherine Carpenter, Frederick Ochieng’

**Affiliations:** 1 Vanderbilt University Institute for Global Health and Department of Health Policy, Vanderbilt University Medical Center, Nashville, Tennessee, USA; 2 Lwala Community Alliance, Nashville, TN, USA; 3 Department of Pediatrics, Vanderbilt University Medical Center, Nashville, TN, USA; 4 School of Public Health, Tulane University, New Orleans, LA, USA; 5 Lwala Community Alliance, Migori County, Kenya; 6 Last Mile Health, Boston, MA, USA; 7 Vanderbilt University, Nashville, TN, USA

**Keywords:** global health partnerships, non-governmental organizations, academic–NGO collaboration, Brocher Declaration, community health, Kenya, capacity strengthening, short-term experiences in global health (STEGHs), bidirectional learning, health systems strengthening

## Abstract

*Background:* Global health partnerships between academic institutions and non-governmental organizations (NGOs) are increasingly recognized as essential mechanisms for strengthening health systems in low-resource settings. Despite their proliferation, few partnerships demonstrate long-term, equitable collaboration grounded in established ethical frameworks.

*Objectives:* To describe and evaluate a 20-year partnership between Lwala Community Alliance, a community-based NGO in western Kenya, and Vanderbilt University in the United States, using the Brocher Declaration principles as an evaluative framework for ethical, sustainable, and mutually beneficial global health collaboration.

*Methods:* We employed a qualitative descriptive case study design, drawing on internal documentation, program records, and publicly available data. The partnership was analyzed across three interdependent pillars—education and training, research, and service—and assessed against each of the six Brocher Declaration principles: mutual partnership with bidirectional learning; empowered host country and community defining needs; sustainable programs and capacity building; compliance with applicable laws and ethical standards; humility and cultural sensitivity; and accountability for actions.

*Findings:* Over 20 years, the partnership engaged 57 graduate students in 33 discrete projects and yielded 12 peer-reviewed publications from a longitudinal household survey encompassing more than 20,000 households. Key outcomes include a reduction in under-five mortality from 105 to 29.5 deaths per 1000 live births, policy reforms formalizing community health worker compensation and digitization across Kenya, and sustained bidirectional capacity strengthening. Adherence to Brocher Declaration principles, including community-led project design, ethical oversight through Kenyan review boards, and structured financial compensation to the host partner, was central to the partnership’s longevity and impact.

*Conclusions:* This case illustrates how the Brocher Declaration can be operationalized to evaluate and strengthen long-term academic–NGO collaborations. The Lwala–Vanderbilt model offers a replicable framework for ethical, impactful, and mutually reinforcing global health partnerships that advance health equity in low-resource settings.

## Background

Global health partnerships have long been viewed as an effective approach to strengthen capacity in low-resourced healthcare systems [1–4]. The World Health Organization (WHO) describes partnerships for health development as a way to “bring together a set of actors for the common goal of improving the health of populations based on mutually agreed roles and principles” [1, 5]. Successful health partnerships can enhance and leverage resources to increase impact on health systems at the local, regional, and national levels. Partners in these activities have often included governments, international organizations, non-governmental organizations (NGOs), local community groups, and academic institutions. The WHO’s definition focuses on the balance of power and alignment of values [1, 5]. Brinkerhoff et al. suggest that the definition of partnerships incorporate two dimensions: mutuality and organization identity [6–8]. These two dimensions separate partnerships from other types of relationships. Mutuality refers to how the relationship advances a cause shared by partners [6]. Organization identity captures the unique traits each partner brings to the relationship, with a focus on how the partner can maintain their mission and values during long-term collaborations [6]. Furthermore, the term “partner” suggests that each party has shared rights and responsibilities, meaning they will obtain mutual benefits from their joint work rather than a one-way transfer of information or skills. This can be imagined in many ways, leaning on the strengths of organizations and what they are able to offer their partners.

Although the earliest formal partnerships in global health were often between religious missionaries and local communities or bilateral government agencies and recipient nation governments, partnerships have expanded exponentially in the last two decades to include international groups, multilateral organizations, foundations, and public–private partnerships. Collaborations between academia and NGOs have increasingly been utilized at the local, regional, and national levels to advance global health and development goals. A study completed by the Center for Strategic and International Studies in 2016 found that the most often perceived benefit between academic institutions and international partners was through education and research collaborations [9]. A common example of this type of collaboration is through exchange programs for medical students and trainees at both institutions [4]. This exchange can be bilateral, but it is most common for high-income trainees to train at host institutions in low-income settings through these partnerships. Much of the existing literature focuses on the benefit of those students who go to partner organizations, and not the host institution’s perspective of the impact of these trainees [10]. Such benefits described in the literature include maximizing global health impact, leadership development, training and mentoring, and health systems strengthening, with emphasis often placed on the sending institution’s benefits [9]. There have been efforts to establish ethical principles or guiding frameworks for these “Short-term Experiences in Global Health” (STEGHs) [11, 12]. In 2022, motivated by concerns around STEGHs and previous guidelines for engagement, the Advocacy for Global Health Partnerships was established. This coalition created the Brocher Declaration with six principles to guide ethical, sustainable, and practical collaboration in the field (Table 1) [13].

**Table 1:** Brocher Declaration principles.

1. Mutual partnership with bidirectional input and learning
2. Empowered host country and community define needs and activities
3. Sustainable programs and capacity building
4. Compliance with applicable laws, ethical standards, and code of conduct
5. Humility, cultural sensitivity, and respect for all involved
6. Accountability for actions

While numerous case studies of successful partnerships highlight their successes and outline lessons learned, success is not clearly defined through indicators or metrics. Additionally, many partnerships have not displayed long-term results from collaboration. Although information on how to develop successful partnerships is plentiful, innovation in development and retention of successful partnerships is lacking throughout current literature. In this reflective narrative paper, we present a model for effective partnership between Lwala Community Alliance, an NGO in western Kenya, and Vanderbilt University, an academic institution in the United States, utilizing principles from the Brocher Declaration. While this partnership predates the declaration, we argue that it exemplifies these principles. By focusing on three primary areas of collaboration—education, research, and service—we highlight lessons learned from this 20-year partnership. In reviewing the partnership and applying the framework for this case report, we utilized internal or publicly available data and reports to gather information on our collaborative education, research, and service projects. Informed consent and ethical approval were not sought because no individual-level data were used.

## History and Context

The Lwala Community Alliance (Lwala) was established in 2007 as a community-based health organization in western Kenya. Lwala is in Migori County, one of Kenya’s 47 administrative counties, which historically underperformed on many health metrics. In 2014, Migori recorded the highest under-five mortality rate in the country, at 82 deaths per 1000 live births, and childhood immunization coverage lagged well below national averages [14]. HIV prevalence was more than twice the national rate [15]. Against this challenging backdrop, Lwala achieved substantial early success: by 2017, under-five mortality in its catchment area had declined to 29.5 per 1000 live births, reflecting significant improvements in access to and quality of care [16].

Lwala was founded by Kenyan physicians, Drs. Milton and Fred Ochieng’, whose journey was chronicled in the documentary *Sons of Lwala*. Because of their academic promise and their parents’ support, the brothers earned scholarships to Dartmouth College and subsequently attended Vanderbilt University School of Medicine. During their medical training in the United States, both of their parents died of AIDS-related complications, a tragedy that inspired the brothers to improve healthcare access in their home community. As part of Vanderbilt’s Emphasis research program, Milton developed a blueprint for a rural health clinic in 2005. Two years later, with guidance from Vanderbilt faculty and substantial grassroots fundraising, the brothers opened the Lwala Community Health Center, which has since evolved into a fully functional hospital entirely staffed by Kenyan clinicians.

Over time, Lwala expanded beyond facility-based care to implement a comprehensive community-led health model that integrates primary care delivery, community health worker (CHW) networks, and health systems strengthening. Through close collaboration with the Kenyan Ministry of Health, Lwala helped professionalize CHWs by advocating for standardized training, supervision, and remuneration. Today, the organization supports more than 9000 CHWs across 3 counties and contributes to improved emergency obstetric and newborn care in over 40 facilities across 6 counties. As Lwala’s operational scale and evidence base have grown, so too has its influence in national and subnational health policy. The organization now serves as a technical advisor to national and county health authorities while maintaining close academic collaboration with Vanderbilt University’s Institute for Global Health and a US-based support team in Nashville.

Vanderbilt University has played a pivotal role throughout Lwala’s evolution, providing technical expertise, student engagement, and early financial support. The partnership originated as a student-led initiative channeled through Vanderbilt’s alumni and development offices, where early donations were managed on behalf of Lwala. Lwala has since become an independent, Kenyan-led NGO employing 183 staff in Kenya and 7 in the United States. The partnership has matured into a model of reciprocal learning and shared benefit, characterized by co-designed projects, joint research initiatives, and aligned institutional priorities that reflect the evolving ethos of equitable global health engagement.

## Model: Education and Training, Research, and Service

Lwala and Vanderbilt University have engaged in partnership through three key areas, Education and Training, Research, and Service. Together, these areas have strengthened the community, healthcare delivery, and professional capacities through community-driven, evidence-based approaches.

### Education and training

Over the past decade, the Lwala–Vanderbilt partnership has strategically engaged graduate-level students across disciplines, including medicine, public health, education, and the social sciences, in service-learning and implementation research that advances both academic training and community health impact. Through carefully structured practicum placements, faculty-mentored field experiences, and co-designed research projects, the partnership has served as a model for integrating graduate education with community-identified priorities in a low-resource setting. Between 2011 and 2025, a total of 57 graduate students have completed formal placements or immersive experiences at Lwala, contributing to 33 discrete projects and 9 peer-reviewed publications. These initiatives illustrate how structured, reciprocal academic–NGO collaborations can foster both skill development for students and sustainable programmatic benefits for local organizations.

One key avenue for student training engagement is Vanderbilt University School of Medicine’s Global Health Immersion Course, a four-week clinical and service-learning experience offered to third- and fourth-year medical students. Since 2011, Lwala has hosted 37 Vanderbilt medical students through this course, which introduces participants to the clinical and public health dimensions of care delivery in resource-limited settings. The curriculum, jointly developed by Vanderbilt and Lwala faculty, emphasizes cultural humility, ethical engagement, and locally led learning. Students complete pre-departure training in cross-cultural communication and health systems context, followed by on-site clinical mentorship under Lwala’s Kenyan health professionals. Each student undertakes a capstone project selected by Lwala leadership to address a defined organizational need, such as improving data management systems or strengthening maternal health counseling tools. These projects have generated actionable outputs for Lwala’s programs and highlighted innovations in community-based healthcare delivery. Weekly reflective mentoring sessions with Vanderbilt faculty further reinforce ethical practice, self-awareness, and applied problem-solving during the rotation, ensuring the experience remains educationally rigorous and contextually responsive.

The partnership has also served as a field training site for students from Vanderbilt’s Master of Public Health (MPH) program and the Peabody College of Education and Human Development, hosting 18 graduate students for two- to three-month practicum placements since 2012. These students have collaborated with Lwala teams on projects encompassing program evaluation, data analytics, monitoring and evaluation (M&E) system design, and the development of educational interventions. These projects have strengthened staff capacity, directly improved programming, and led to publications, which are highlighted in the “Research” section below. Each practicum culminates in a tangible deliverable such as a new data collection tool, training curriculum, or operational framework that Lwala can integrate into its ongoing programs. One illustrative example involved a 2013 MPH student who trained Lwala staff in geographic information systems (GIS) mapping and documented community water sources. Six months later, Lwala staff independently applied these skills to trace the source of a local cholera outbreak, demonstrating both the immediate utility of the student’s work and the sustainability of capacity built through the collaboration.

A third stream of engagement links Lwala to courses in the Vanderbilt MPH Program. Vanderbilt course instructors have partnered closely with Lwala, fostering a model of sustained, community-led collaboration that stands apart from typical short-term, student-driven engagements. In many university programs, students are encouraged to select their own nonprofit partners for practicum or capstone projects. While this “student-led” model offers flexibility, it often results in fragmented and transient partnerships. Projects may lack continuity, institutional memory, or meaningful local impact once the student departs. In contrast, Vanderbilt’s partnership with Lwala is intentionally structured around long-term, community-led engagement. Each student project builds upon previous work and lays the foundation for future efforts, creating an iterative process of refinement and shared learning. This continuity strengthens project quality, deepens institutional knowledge, and fosters bidirectional learning, while also mitigating the ethical and operational risks associated with short-term, extractive engagements. The characteristics of this enduring collaboration spanning over a decade between a regional Kenyan health organization and a major US research university make it a relatively unique and instructive model for sustainable global partnerships.

A central component of this collaboration has been the “Essential Skills in Global Health” course. Since 2014, Lwala has served as an annual partner for this project-based course, which uses real-world collaboration to help students develop essential competencies in global health practice*.* Crucially, projects are identified by community partners rather than by faculty or students, ensuring that efforts are locally driven and contextually relevant. To date, 13 projects have been implemented with Lwala through this course, covering diverse areas such as early childhood development, organizational culture in health facilities, and men’s attitudes and practices toward gender equality and women’s reproductive health.

Through this model, students gain hands-on experience in applied research and program design, while Lwala benefits from new analytical tools, data insights, and perspectives that advance its ongoing initiatives. The result is a mutually reinforcing partnership—one that integrates education, research, and service while demonstrating how academic institutions can engage ethically and effectively with community-based organizations in global health.

Taken together, these educational collaborations illustrate how thoughtfully structured, reciprocal partnerships can bridge academic learning with community-driven implementation research. The Lwala–Vanderbilt model demonstrates that when global health training programs prioritize ethical engagement, co-design, and measurable outcomes, graduate education can simultaneously advance student competencies and strengthen local health systems—fulfilling the dual mandate of education and service central to sustainable global health partnerships.

### Research

Research serves as a cornerstone of effective global health programming and policy development, yet organizational capacity for research within NGOs remains limited, particularly in resource-constrained settings [2]. Strengthening the ability of local NGOs to conduct and apply research is essential to ensuring that evidence flows upstream from community realities to national and international policy agendas [17, 18]. Partnerships between academic institutions and NGOs can play a pivotal role in this process: while NGOs bring contextual knowledge, community trust, and implementation reach, academic partners contribute methodological expertise, analytical rigor, and access to research infrastructure [2, 19]. When grounded in equity and co-creation, such collaborations can enhance research capacity as well as service delivery, institutional development, and policy influence [17, 20].

Within this framework, Lwala and Vanderbilt University have built a robust and sustained research partnership that has generated actionable evidence for both community programs and the broader global health field. Lwala has also invested in a team of researchers locally who have been integral to this effort. Most notable among these projects is a large, repeated cross-sectional household survey to quantify key health metrics [21]. These findings formed the basis for the longitudinal household survey and helped build evidence for Lwala’s current expansion. The initial survey in 2017 was designed to capture health data about households in Lwala’s original catchment area. That year, a dual degree Vanderbilt MPH/MD student conducted a study of under-five mortality in Lwala, which was published in 2018 in the journal, *PLOS One* [16]. Results showed that prior to a 2010 intervention by Lwala, 105 children under 5 died per 1,000 live births. By 2017, that rate dropped to 29.5 deaths per 1,000 live births. Lwala’s rates outperformed the reported under-five mortality rates for the region, Nyanza Province (82 per 1,000), and for Kenya as a whole (52 per 1,000) [14].

Since its inception, the household survey has evolved into a 10-year longitudinal evaluation encompassing more than 20,000 households. This project has grown alongside work with the county government to scale health and development programming and has provided both valuable baseline data prior to program implementation and a growing body of evidence for the efficacy of Lwala’s programming. Furthermore, the robust dataset has formed the basis of 12 academic publications [16, 21–31] with at least 15 more in various phases of development and serves as a training platform for emerging researchers. Between 2005 and 2025, 25 Vanderbilt trainees, including medical, MPH, and doctoral students, have conducted research projects using Lwala’s data, all in collaboration with Lwala’s Kenyan research team, which has grown from 1 staff member to 7 over this period. Using the repository of longitudinal household survey data for collaborative research is mutually beneficial for Lwala staff and Vanderbilt partners, as well as the wider community. Publications arising from this household study contribute meaningfully to regional literature, addressing critical research gaps while benefiting Kenyan students and the broader academic community. The reciprocal benefits of this research are reflected in changes in authorship as well. In a recent journal supplement (with a series of articles drawn from the household survey), 12 out of 15 have Kenyan first authors, and 2 of the 7 mentors guiding these papers are professors from local Rongo University. Building on the successful model of engagement established with Vanderbilt, Lwala is now pursuing similar partnerships with Strathmore University and Rongo University to collaborate with local students on research and training initiatives. In selecting a research question based on the type of survey data collected, the study remains focused on Lwala’s central research mission rather than the visitors’ interests. This model minimizes participant fatigue by using existing data and ensures that findings are rapidly translated into programmatic decision-making.

Beyond the household survey, the partnership has contributed to policy-relevant research on CHW effectiveness [32] and has been involved in international research collaborations around CHW compensation and service provision [33, 34]. This research has directly addressed knowledge gaps identified in WHO guidelines [35] and has contributed to county and national policy change in Kenya. After years of advocacy from Lwala and its partners, the Kenyan government passed a series of sweeping reforms in late 2023. First, a landmark Primary Health Care (PHC) Act formalized the role of CHWs in the health system and codified their right to payment. CHWs in 45 of Kenya’s 47 counties are now being paid, and work is ongoing to strengthen payment mechanisms and consistency. Second, the national government launched the electronic Community Information System (eCHIS), a new digital platform developed to digitize Kenya’s community health workforce and improve decision-making. It has since been rolled out to 124,000 CHWs across Kenya. Finally, the national government distributed standardized kits to equip CHWs with supplies and medications needed for daily services. Collectively, these reforms mark transformative progress toward universal health coverage and reflect the direct policy influence of evidence generated through academic–NGO collaboration.

The Lwala–Vanderbilt partnership also fosters iterative, student-led research continuity, where successive projects (identified as community priorities) build cumulatively on previous work. For instance, between 2007 and 2011, three Vanderbilt medical students conducted a coordinated series of studies on intestinal helminth infections in children and adults. Each project refined research questions and methodologies from the prior study, creating a quasi-longitudinal dataset that informed targeted community interventions. Findings from this research series resulted in three peer-reviewed publications co-authored by both Lwala and Vanderbilt researchers, illustrating the value of continuity and co-mentorship in capacity building and scientific productivity [36–38].

While ethical review at the beginning of the partnership was only done at Vanderbilt University, Lwala-based research is now approved first by a local ethics review board at Kenyan universities, and appropriate research licenses are obtained from the Kenyan government. Data monitoring boards monitor all projects for negative outcomes, and these are immediately reported to appropriate boards.

Taken together, these efforts demonstrate that research partnerships grounded in co-design, mutual capacity strengthening, and community relevance can move beyond traditional academic outputs to inform policy reform, health system strengthening, and local ownership of data. The Lwala–Vanderbilt experience underscores how research can function not only as a scholarly pursuit but as a mechanism for sustainable impact, linking evidence generation directly to the goals of education, service, and equitable global health development.

### Service

Service and institutional engagement have been foundational to the Lwala–Vanderbilt partnership, translating academic collaboration into sustained community impact and reciprocal institutional growth. Since Lwala’s inception, Vanderbilt students, faculty, and staff have applied their skills and enthusiasm to support the organization’s mission to achieve health equity in rural Kenya. This service extends directly to local researchers and faculty, who receive structured mentorship throughout the research process—from study design through manuscript development—enabling them to translate that guidance into their own first-authored publications. This mentorship builds lasting capacity: as local researchers grow their skills, they in turn become mentors themselves, extending the same guidance to students and junior colleagues at their institutions. Through these engagements, academic expertise has been leveraged to design practical solutions for local challenges while simultaneously advancing student learning in applied global health practice, creating a cycle of growth that strengthens research capacity on both sides of the partnership.

Over the course of the partnership, Vanderbilt students have developed a suite of implementation tools including survey instruments, M&E frameworks, CHW capacity assessment tools, and program scorecards, all of which have strengthened Lwala’s systems for data-driven decision-making and performance management.

A particularly impactful avenue of engagement has been Vanderbilt’s Global Health Case Competition, hosted by the Vanderbilt Institute for Global Health (VIGH). In 2019 and 2023, Lwala staff were invited to co-develop case challenges grounded in community-identified priorities [39]. The 2019 case, *Improving Non-Communicable Disease Care through Expansion of Lwala’s Health Services in Rural Kenya*, focused on addressing rising chronic disease burdens in underserved settings. The 2023 case, *Addressing Drinking Water Quality Issues in Rural Migori County, Kenya*, built upon an MPH student’s field research testing community water sources for contaminants. In each competition, Vanderbilt students engaged directly with Lwala staff to understand contextual barriers, develop innovative solutions, and present their recommendations to panels that included Lwala representatives and board members. These interactions provided authentic exposure to global health systems and implementation realities while generating actionable insights for Lwala. As a direct outcome, Lwala launched new programming targeting non-communicable diseases, including the creation of patient support groups, designated clinic days, and the deployment of community-based patient ambassadors to improve prevention, care continuity, and community mobilization.

Clinical collaboration has also been central to service learning within the partnership. Vanderbilt medical students participating in the global health clinical immersion course complete rotations at Lwala Community Hospital, where they design and deliver Continuing Medical Education (CME) sessions as part of their capstone projects [40]. Guided by Lwala clinical supervisors, students identify training needs through direct observation and clinical participation, resulting in CME sessions on topics such as neonatal resuscitation, management of pediatric pneumonia and tuberculosis, HIV testing and counseling, preterm birth, and health systems delivery. These sessions provide reciprocal learning opportunities, allowing students to refine their teaching and clinical reasoning skills while contributing to the professional development of Lwala’s healthcare staff. It’s worth noting that US students don’t “train” Kenyan healthcare staff—rather, the relationship is collaborative: they share ideas, ask questions, conduct literature reviews, and occasionally introduce new tools. This exchange runs both ways, as the opportunity to teach and mentor these students also helps expand the skills of Lwala staff themselves, reinforcing the reciprocal nature of the partnership.

The partnership has also supported capacity-building projects that help the organization strengthen staff skills and improve health outcomes. In 2017, a group of medical students worked with Lwala staff to develop a Clinical Quality Assessment tool, designed to identify gaps in protocol adherence and staff knowledge. The organization has used this tool to target future training and mentorship efforts, adapting it over time to meet evolving needs. In 2018, another student conducted a quality assessment of postpartum hemorrhage (PPH) care at Lwala and neighboring government facilities using a validated tool. She shared her findings with Lwala, which led directly to the creation of a new clinical training program on the subject. In 2021, a student supported a study tracking the implementation of Helping Babies Breathe at Lwala and other government facilities. The research led to meaningful improvements in the organization’s training approach.

Between 2013 and 2019, the partnership also leveraged technology to facilitate virtual clinical case conferences, connecting Lwala’s clinicians with Vanderbilt medical experts across specialties including infectious diseases, pediatrics, and obstetrics. These monthly discussions provided real-time mentorship and specialty consultation during Lwala’s transition from a community health center to a full-service hospital. This support mostly took the form of case reviews, where the team sought specialized opinions or clinical input. It was particularly valuable during periods when Lwala lacked a Medical Officer on staff, though its relevance has endured—any clinical team stands to gain from opportunities to build skills and deepen knowledge in specialized areas of medicine. Through VIGH, Lwala has access to some of the world’s leading specialized clinicians, spanning fields such as HIV, sickle cell disease, oncology, and cardiology. While the impact of this support has not been formally measured, its value to the clinical team has been consistently evident.

Beyond this technical support, the Vanderbilt community has mobilized substantial financial and in-kind resources through fundraising events, direct donations, and sustained institutional funding. Notably, this early backing from Vanderbilt and its broader community helped build Lwala’s credibility and reputation, proving pivotal in attracting outside funding. Support from the Vanderbilt community remains particularly valuable because it comes bundled with capacity building, validation, and research and learning contributions that extend well beyond direct financial resources. This institutional engagement also extends to governance: eight Vanderbilt affiliates—four faculty and four alumni—currently serve on Lwala’s Board of Directors, while two Vanderbilt faculty members sit on the Advisory Board, ensuring strategic alignment and continuity between the two institutions.

Importantly, the financial model underlying this partnership represents a replicable best practice for other university–NGO collaborations. Since 2008, an annual unrestricted grant from Vanderbilt has provided flexible support for Lwala’s operational and research activities, including trainee supervision, infrastructure costs, and community engagement. This funding, complemented by in-kind contributions such as shared office space, offsets the real labor, time, and logistical investments required of Lwala staff to host students and facilitate research. Too often, partnerships rely on in-kind contributions from local organizations without compensation, creating structural inequities. By contrast, Vanderbilt’s approach explicitly acknowledges and funds the effort expended by its partner, setting a precedent for ethical cost-sharing and institutional accountability in global health education and research. Future university partnerships seeking to model this approach should consider incorporating formal financial arrangements that value the local partner’s expertise, ensure equitable resource flow, and promote long-term sustainability. While Vanderbilt once represented the majority of Lwala’s overall funding, Lwala has since diversified its funding base, and this source now represents a smaller share of the organization’s total support. Additionally, Lwala as an organization is not dependent on Vanderbilt. While student opportunities do depend on the partnership, this dependency does not put Lwala’s other work at risk. Both partners benefit from the relationship, and Lwala’s contributions actively enhance Vanderbilt’s offering to its students. Lwala provides a valuable service; that service strengthens what Vanderbilt can offer its students; and student tuition, Vanderbilt’s endowment, and government grants represent sustainable, diversified funding streams that stand independent of the partnership itself.

A critical factor contributing to the longevity and success of the Lwala–Vanderbilt partnership has been the continuity of leadership and key relationships on both sides. From its inception, Lwala’s founding and early leadership included 2 Vanderbilt alumni, and the organization has undergone only 1 executive leadership transition in its 20-year history, an uncommon level of stability among community-based NGOs. This continuity has allowed for sustained trust, institutional memory, and alignment of shared goals over time.

Similarly, at Vanderbilt, the ongoing engagement of key faculty and staff, some of whom have been involved for more than a decade, has provided a foundation of relational consistency and organizational learning. These long-term connections have facilitated the ability to build upon prior projects, navigate challenges collaboratively, and maintain mutual accountability. Beyond its functional success, the partnership appears to have served as a source of professional identity and pride for those involved, fostering a sense of belonging and purpose that distinguishes it from the more transactional, short-term engagements often seen in university–NGO collaborations. This sustained, trust-based continuity underscores the importance of relational stability as a core determinant of partnership resilience and impact.

Collectively, these initiatives illustrate how sustained, ethically grounded partnerships can integrate education, research, and service to promote both institutional strengthening and community health outcomes. The Lwala–Vanderbilt collaboration demonstrates that equitable global health engagement extends beyond short-term student experiences to long-term capacity exchange and is one that builds shared expertise, mutual accountability, and measurable community benefit.

## Discussion

We believe the Brocher Declaration principles provide a structure and an ongoing evaluation tool for successful global health partnerships. This is not a partnership between two universities, where a bilateral exchange and engagement of students would be the natural benchmark. Rather, this is a partnership between a Kenyan NGO and a US academic institution, so reciprocal benefits flow through channels appropriate to each partner’s role rather than through a mirrored exchange of students in each direction. Bidirectionality here is evidenced in what each side receives, not in structural symmetry. The long-standing partnership between Lwala and Vanderbilt University is based on shared values, driven by community-identified needs, aligned with the goals of the partner organization, and focused on mutual benefit. Because priority-setting is conducted by both parties through open and transparent conversation, there is little space for mission creep, which is often seen in these types of partnerships. We outline below how the principles were used to characterize our partnership (Table 2). It is important to note that this alignment reflects the partnership’s current state, reached through years of iterative adjustment rather than an original design. As detailed in Lessons Learned below, several of these principles—particularly shared governance and sustainable capacity building—were not consistently met in the partnership’s earlier years.

**Table 2:** Application of Brocher Declaration Principles to the partnership between Lwala and Vanderbilt.

1. *Mutual partnership with bidirectional input and learning* Education: Curricula co-designed for bidirectional learning Research: Authors from Lwala and Vanderbilt included on all projects to ensure mutual skill development and attribution Service: Mutual identification of needs informs projects
2. *Empowered host country and community define needs and activities* Education: Educational projects selected by Lwala to allow use of student efforts to remedy identified problem Research: Research priorities directly established by Lwala and county government Service: Needs are defined by Lwala, solutions are co-developed, and implementation is led by Lwala
3. *Sustainable programs and capacity building* Education: A long-term nonprofit–university partnership helps facilitate and support educational programs. Lwala integrated into multiple Vanderbilt classes and degree programs providing infrastructure for such a partnership Research: Projects transferred to Lwala employees for capacity strengthening and integration into normal operations Service: CME courses carried out by Vanderbilt students and faculty support long-term capacity strengthening of Lwala clinicians who provide direct care
4. *Compliance with applicable laws, ethical standards, and code of conduct* Education: Students undergo cultural competence and ethical training prior to trip to Lwala Research: All research is approved by a local Kenyan ethical review board/IRB, and appropriate research licenses are obtained from the Kenyan government Service: Supervision by Lwala staff on the ground ensures ongoing compliance with ethical standards and local laws by visiting students and faculty, in addition to written agreements
5. *Humility, cultural sensitivity, and respect for all involved* Education: Pre-travel curriculum is designed to educate students about local history and customs as well as to encourage humility Research: Lwala programming (non-research) staff included in research to provide context and potential applications Service: Opportunities originate from Lwala employees and are grounded in a capacity strengthening approach
6. *Accountability for actions* Education: Bidirectional feedback allows for open dialog regarding programs and particular students/staff Research: Data sharing agreements, ethical/IRB oversight, board oversight, and active inclusion of communities and local government in research initiatives Service: Students complete a post-service survey, Lwala regularly solicits feedback from communities through dialogues, anonymous reporting platforms, and household surveys

Using a framework such as the Brocher Declaration for continuous assessment can be particularly valuable, as the nature and structure of partnerships often evolve over time. Many university courses allow students to select their own nonprofit partners for field experiences. While this “student-led” model may offer convenience, it has inherent limitations: short-term engagements frequently result in fragmented projects with limited continuity or sustained impact. In contrast, Vanderbilt’s collaboration with Lwala has been structured around a long-term, community-led model, where current student projects build upon prior work and serve as the foundation for future efforts. This continuity reduces fragmentation, facilitates iterative improvement, promotes bidirectional learning, and mitigates the ethical and operational risks associated with short-term interventions. The durability and mutuality of this partnership are relatively unique for a collaboration between a regional health organization and a major research university.

## Lessons Learned

Over the course of the partnership, several challenges have emerged, providing opportunities for reflection and improvement. The original financial oversight by Vanderbilt offered limited transparency and constrained Lwala’s agency in donor relations, conditions misaligned with the mutuality and shared governance principles articulated in the Brocher Declaration. Over time, however, both institutions learned valuable lessons about equity, autonomy, and accountability in cross-institutional collaboration.

Language and cultural differences have sometimes limited the effectiveness of student–clinician interactions, and incoming students have occasionally lacked sufficient familiarity with local medical standards, including guidelines established by the Kenyan Ministry of Health. Additionally, although communication is constant and the partnership is long-standing, the limited duration of student rotations has, at times, constrained the execution of larger or more complex projects, leaving some initiatives unfinished and, in certain cases, increasing the workload for local staff. Strategies to address these challenges have included enhanced pre-departure training, structured handoffs between successive student cohorts, and intentional project design that emphasizes continuity. In the initial years, undergraduate students from multiple universities participated in an ad hoc manner, which proved difficult to coordinate and limited continuity. These experiences informed a shift toward graduate-level, single-university cohorts and the implementation of regular check-ins via Skype or Zoom during immersion rotations. These virtual meetings provided a structured space for students to discuss challenges, allowed supervisors to address concerns from Lwala staff in real time, and fostered a culture of shared learning and continuous improvement. By embracing an iterative approach, the partnership was able to transform early logistical and experiential “failures” into opportunities for enhancing supervision, communication, and mutual benefit. These adaptations highlight how a reflective, iterative partnership model can transform early obstacles into opportunities to strengthen both student learning and local organizational impact.

## Limitations

We acknowledge that this reflective narrative is not without bias. Each author has been actively engaged with Lwala and Vanderbilt for several years and knows the partnership well. Despite this bias, we believe that the examples we selected are representative of the partnership and were among dozens of similar examples that could have been described. Additionally, the Brocher Declaration was developed as a framework to guide short-term global health experiences, but we have adapted it to a long-term global health partnership. However, we believe it provides a helpful framework to ensure balance in the evaluation of the partnership. We have added additional context regarding long-term outcomes throughout the manuscript.

## Reflections and Implications

As global health partnerships continue to evolve, the need for ethical, sustainable, and mutually beneficial frameworks to guide work in the field has become increasingly pressing. The Brocher Declaration principles offer an essential normative foundation for equitable collaboration, particularly for STEGH; however, limited empirical evidence exists on how these principles can be systematically operationalized within long-term institutional partnerships.

Drawing on a 20-year collaboration between academic and NGO partners engaged in education, research, and service in global health, we present an applied model that translates the Brocher framework into a set of actionable strategies and evaluative criteria, which appear useful for long-term partnerships. Our model emphasizes shared governance, bidirectional capacity strengthening, contextual responsiveness, and longitudinal assessment of partnership outcomes. By integrating principles of equity and reciprocity into partnership design, implementation, and evaluation, this model illustrates a pathway for institutions to move from values-based commitments to measurable practice.

Findings highlight how intentional alignment with the Brocher Declaration can advance institutional accountability, foster adaptive learning, and ensure sustained mutual benefit. This work contributes to the growing literature on global health partnership ethics by offering a pragmatic approach to assessing and enhancing partnership efficacy over time.

It is worth noting that this model was not achieved without setbacks common to high-income–low-income institutional collaborations. Early financial oversight structures limited Lwala’s autonomy and transparency in donor relations, short-term student rotations at times left work unfinished and increased local staff burden, and gaps in cultural and clinical-context preparation among incoming students periodically strained service delivery. These are not incidental footnotes but recurring risks inherent to this partnership type, and we believe the Brocher Declaration’s value lies precisely in surfacing and correcting them over time—rather than in certifying a partnership as static or complete. Institutions considering a similar model should expect, and plan for, this iterative process rather than assume immediate alignment across all six principles.

## Data Access and Writing Verification

All authors confirm that they had access to the data and information presented in this manuscript and played an active role in its writing, revision, and final approval.
